# The recording of personality strengths: An analysis of the impact of positive personality features on the long‐term outcome of common mental disorders

**DOI:** 10.1002/pmh.1548

**Published:** 2022-05-09

**Authors:** Min Yang, Peter Tyrer, Helen Tyrer

**Affiliations:** ^1^ West China School of Public Health, Sichuan University West China Medical Centre Sichuan University Chengdu China; ^2^ Faculty of Health, Art and Design Swinburne University of Technology Melbourne Australia; ^3^ Division of Psychiatry, Department of Brain Sciences Imperial College London UK; ^4^ Lincolnshire Partnership NHS Foundation Trust Lincoln UK

## Abstract

Although personality strengths are assessed frequently in occupational and managerial settings and in children, they have been less used in studies of personality disorder. The aim of this study is to examine the impact of a measure of personality strengths derived from the comprehensive version of the Personality Assessment Schedule (CPAS) (i.e., positive and reinforcing traits) on clinical symptoms and functioning. Eighty‐nine patients with anxiety and depression seen at the 30‐year follow‐up point in a cohort study (Nottingham Study of Neurotic Disorder) were administered the Comprehensive version of the PAS (CPAS). A factor analysis of the results determined the main groupings and their impact on long‐term outcomes as well as their association with change of outcomes over 30 years. Five positive factors (strengths), forceful considerateness, emotional toughness, cautiousness, independence and discernment accounted for 67.2% of the variance using both Varimax and Promax rotations. Low positive scores were strongly associated with suicide attempts, moderate/severe personality disorder, cothymia (mixed anxiety‐depression), greater symptomatology and poor social function. High scores were protective of serious pathology and particularly effective in inhibiting suicidal behaviour. The promotion of personality strengths may be of value in preventing suicidal behaviour and helping pro‐social change in those with personality disturbance.

## INTRODUCTION

Although there has been considerable interest in personality or character strengths in occupational psychology and personality theory (Peterson & Seligman, 2004), and also in children (Goodman, 2001), this has not extended far into research with people who have personality disorder. Although there have been many who have promoted the assessment of positive personality characteristics when assessing people with personality disorder (Clarkin et al., 2018; McAdams & Pals, 2006; Sadeq & Molinari, 2018), this has not been converted into studies involving formal measurement.

In a long‐term follow‐up study of common mental disorders (Nottingham Study of Neurotic Disorder), an assessment of personality strengths was felt to be valuable. A scale had been established in 1982, The Comprehensive Personality Assessment Schedule, that included such an assessment, but this was never published and not used originally in the Nottingham Study because it was felt to be too long for a multi‐assessment interview. No previous psychometric data had been determined for this scale.

## METHOD

### Original randomised trial

The Nottingham Study of Neurotic Disorder was initiated in October 1983, and its general methodology is described in earlier papers (Tyrer et al., 1988, 1990). The participants were 210 patients, first seen by PT and his clinical team in general practice psychiatric clinics in Nottingham between 1983 and 1987, who were at the time of assessment required to be on no psychiatric treatment and to have no major mental illness. The status of these patients is best considered as midway between primary and secondary care (Tyrer, 1984; Williams & Balestrieri, 1989), with fewer than those in out‐patient clinics having had previous treatment.

The participants, all with a DSM‐III diagnosis of generalised anxiety disorder, dysthymic or panic disorder determined after a structured interview (Spitzer & Williams, 1983), were involved in a randomised trial initially, with allocation to drug treatment, cognitive behaviour therapy and self‐help for the first 10 weeks of the study. Ten of the patients did not complete all baseline data and are not included here.

### Clinical assessments

At baseline, those satisfying a DSM‐III diagnosis of one or more of the three neurotic disorders were made, with total psychopathology determined with Comprehensive Psychopathological Rating Scale (CPRS) (Åsberg et al., 1978), an interview that assesses both current symptomatology and includes a mental state evaluation. Self‐rated anxiety and depressive symptoms were made using the anxiety and depression sections of the Hospital Anxiety and Depression Scale (HADS) (Zigmond & Snaith, 1983) and observer assessments of anxiety and depression made using the Brief Scale for Anxiety (BAS) (Tyrer et al., 1984) and the Montgomery and Åsberg Depression Rating Scale (MADRAS) (Montgomery & Åsberg, 1979). Both of these are derivations of the CPRS. All these clinical assessments were made again at 2, 4, 6, 10, 16, 32, 52 and 104 weeks and again at 5, 12 and 30 years after enrolment into the randomised trial. The reliability of observer assessments was determined by training of all investigators at baseline, and raters were not approved until kappa agreements of 0.65 were achieved across all personality raters after training with PT and 0.8 levels for individual assessments.

All the assessments, apart from those at 5 years, were made at face‐to‐face interviews by assessors unaware of initial diagnosis or allocation. At 5 years, the assessment of services and treatment was made from case notes alone (Seivewright et al., 1998). At 12 and 30 years, two additional assessments were made, the first of social function using the Social Functioning Questionnaire (SFQ) (Tyrer et al., 2005) and the Neurotic Disorder Outcome Scale (NDOS) (Tyrer et al., 2004), a composite measure of clinical, service and functional outcomes. Higher SFQ and NDOS scores indicate lower social function and worse neurotic disorder outcome.

The primary outcome at each major follow‐up point was the dichotomous absence or presence of a DSM diagnosis, excluding minor conditions such as simple phobia and adjustment disorders.

### The general neurotic syndrome: A Galenic syndrome

An additional assessment was made of the general neurotic syndrome, a combined syndrome of mixed anxiety‐depression and dependent and obsessional personality features (Andrews et al., 1990; Tyrer, 1985, 1989; Tyrer et al., 1992), using the General Neurotic Syndrome Scale, in which a score of 4–5 suggests the presence of the syndrome is likely and one of 6 or more indicates the definite presence of the syndrome (Tyrer, 1989; Tyrer et al., 2016), The general neurotic syndrome is one of a number of Galenic syndromes, conditions in which a mental state and personality disorder are so closely entwined they are best considered as a single syndrome (Tyrer et al., 2022), and for the purpose of this research was regarded as a separate entity.

### Personality assessment

Personality assessment was made on four occasions from baseline onwards, using the original Personality Assessment Schedule (PAS) (Tyrer et al., 1979; Tyrer & Alexander, 1979). The changes in personality status over time in this study have been described previously (Yang et al., 2021); they showed that personality status usually changed. The Personality Assessment Schedule scores 24 personality traits on an eight point scale and takes about 20–30 min to complete in a face‐to‐face interview. It shows strong concordance with the new ICD‐11 classification of personality disorder, and the baseline scores have been converted into the four levels of ICD‐11 personality disturbance—personality difficulty, mild, moderate and severe personality disorder (Tyrer et al., 2019) after a full analysis of all data (Tyrer et al., 2014). The Comprehensive Personality Assessment Schedule (CPAS) includes the 24 standard personality traits but also scores their 24 positive equivalents.

At the last follow‐up at 30 years, the CPAS was used to assess both personality disturbance and personality strengths concurrently. As this instrument has to be given in a face‐to‐face interview, only the 30 year data were therefore available for the personality strength component of the schedule. The interviews were carried out with mainly by HT but also a small number by PT with three carried out together to test agreement.

Although the CPAS was only assessed at the 30 year follow‐up, it was felt appropriate to examine its association with clinical symptoms, behaviour and service contacts recorded at both 12 and 30 years, and with the changes of those measures over the period of 18 years. We assumed that any improvement of clinical symptoms, behaviour and reduced service contacts over time could be related to stronger personality strengths by the end of the period.

The hypotheses tested were based on the limited previous evidence of personality strengths being of value in occupational settings (Harzer & Ruch, 2015; Kim et al., 2018). It was felt that the presence of greater personality strengths, irrespective of personality problems, would buttress the person against adversity and, in the context of the Nottingham Study, (a) improve social function, (b) reduce suicidal behaviour and (c) reduce clinical psychopathology.

## STATISTICAL ANALYSIS

This study only included a sub‐cohort of 89 patients who had both survived and were able to be interviewed for the CPAS after 30 years. Out of 210 original patients, 71 had died, and 50 were lost to follow‐up or not interviewed at different time points. There was a non‐significant increase in premature death for those with personality disorder (Tyrer, Tyrer, et al., 2021).

The study focused at the personality strengths of the CPAS. We first performed the exploratory factor analysis (EFA) to summarise the 24 items of CPAS positive items (PASP) by five factors. Both Varimax and Promax rotations generated the same five factors. These factors were further confirmed by clinical experts for their personality natures and collective names. To examine the association between PASP and categorical variables such as suicidal behaviour and service uses at any one time point, means of the different PASP factors were calculated and one‐way analysis of variance used to compare personality strength between levels. The same method was used to test the PASP factor scores among patient groups separated by three elements of personality change over 30 years—no change, change to less personality dysfunction and change for worse dysfunction. To examine the impact of PASP factors on continuous clinical measures including the Social Function Questionnaire (SFQ) (Tyrer et al., 2005), the Neurotic Disorders Outcome Scale (NDOS) (Tyrer et al., 2004) and clinical symptomatology, Pearson's correlation coefficient was used to study the association at 12 and 30 years, respectively. We calculated the difference in SFQ, NDOS and other scales between 12 and 30 years, and its correlation coefficient with PASP factor scores to determine whether improved social function, neurotic disorders and clinical symptomatology were related to a stronger personality strength at later time.

All the above analyses were conducted for each strength factor individually and for the overall PASP score. We used the IBM SPSS 19.0 for the analysis.

## RESULTS

### Analysis of personality strengths

The factor analysis of the data from the CPAS on personality strengths showed it was composed of five elements, termed forceful consideration, emotional toughness, cautiousness, independence and discernment. All showed high inter‐correlation (Table 1).

**TABLE 1:** The five personality strengths factors identified with percentage of variance explained

Factor	Variance explained, %	Nature of personality strengths (from CPAS assessments)	Collective name
1	16.2	Carefulness, placidity, compassion, prudence, maturity	Forceful considerateness
2	32.3	Solidity, self‐esteem, stability, equanimity, openness, self‐sufficiency, resilience	Emotional toughness
3	45.8	Decisiveness, friendliness, thoughtfulness, flexibility, reliability	Cautiousness
4	57.8	Social competence, detachment, self‐directedness, nonconformity, self‐awareness	Independence
5	67.2	Sagacity, self‐confidence	Discernment

These factors were examined individually in the analysis of the Nottingham data set.

### Social function

All personality strengths were strongly correlated with the social function scale at both 12 and 30 years follow‐up, indicating poorer social function was associated with weaker personality strengths (Table 2). The change was defined as the score at 30 years minus the one at 12 years. A negative difference indicated improved social function over the period of 18 years. The mean score of SFQ of the patients was 8.09 (SD 5.63) at 12 years' follow‐up and 7.92 (SD 5.56) at 30 years, an insignificant improvement over the period (paired *t* test *p* = 0.763), and no correlation between the change in SFQ score and personality strengths was demonstrated (Table 2).

**TABLE 2:** Pearson's correlation coefficients between social function and neurotic disorder outcome with five personality strengths

	Personality strengths					
	Forcefulness	Emotional toughness	Cautiousness	Independence	Discernment	Overall
SFQ						
At 12 years (*N* = 89)	−0.545***	−0.475***	−0.557***	−0.381***	−0.338***	−0.546***
At 30 years (*N* = 88)	−0.604***	−0.618***	−0.625***	−0.587***	−0.367***	−0.681***
Change over 18 years	−0.043	−0.140	−0.056	−0.195	−0.016	−0.120
NDOS						
At 12 years (*N* = 89)	−0.490***	−0.469***	−0.564***	−0.379***	−0.274**	−0.528***
At 30 years (*N* = 88)	−0.555***	−0.604***	−0.580***	−0.570***	−0.349**	−0.649***
Change over 18 years	−0.205	−0.285**	−0.165	−0.330**	−0.131	−0.284**

Abbreviations: NDOS, neurotic disorder outcome scale; SFQ, Social function Questionnaires.

** *p* < 0.01.

*** *p* < 0.001.

### Neurotic Disorders Outcome Scale

The relationship between NDOS and personality strengths was similar to that of SFQ at both 12 and 30 year follow‐up (Table 2). However, overall, there was a significant improvement in the scale scores at 30 years with the mean score of NDOS reduced from 2.13 (SD 1.75) at 12 years down to 1.74 (SD 2.21) at 30 years (paired *t* test *p* = 0.047). A greater improvement in the NDOS over the period of 18 years was associated with stronger emotional toughness and independence of individuals (Table 2).

### Distribution by sex and initial DSM diagnosis

The distribution of the personality strengths showed somewhat higher scores for cautiousness in women but few other sex differences. Those of younger age (less than 36 years) had higher strength of forceful consideration, and those with a combined anxiety and depressive diagnosis (cothymia) had fewer personality strengths (Table 3).

**TABLE 3:** Personality strengths by sex, age and DSM diagnosis at baseline

Personality strengths: Mean ± SD						
Characteristics	Forcefulness	Emotional toughness	Cautiousness	Independence	Discernment	Overall
Sex: *N*						
Female: 63	17.8 ± 7.2	21.6 ± 11.4	14.8 ± 7.2	11.3 ± 5.0	4.5 ± 3.1	68.0 ± 27.9
Male: 26	15.2 ± 6.8	19.1 ± 10.6	11.7 ± 6.5	11.7 ± 5.4	3.8 ± 3.1	60.0 ± 25.5
*P* value	0.121	0.333	0.061	0.777	0.321	0.211
Age: *N*						
<36 years	18.1 ± 6.7	21.0 ± 11.9	14.2 ± 7.9	11.6 ± 5.3	4.4 ± 3.1	67.3 ± 28.4
36 years and above	14.9 ± 7.7	20.7 ± 9.4	13.1 ± 5.4	11.1 ± 4.5	4.1 ± 3.2	62.3 ± 23.8
*P* value	**0.043**	0.902	0.488	0.656	0.657	0.419
DSM diagnosis						
Dys: 1	8 ± n/a	12.0 ± n/a	7.0 ± n/a	11.0 ± n/a	2.0 ± n/a	39.0 ± n/a
GAD: 26	18.1 ± 7.5	22.8 ± 11.6	13.7 ± 8.3	12.3 ± 4.8	3.8 ± 3.3	68.5 ± 29.2
Panic: 32	19.6 ± 5.7	23.0 ± 11.3	16.8 ± 6.5	12.4 ± 5.4	5.1 ± 3.1	74.7 ± 26.0
Cothymia: 30	13.8 ± 7.1	17.2 ± 9.9	11.3 ± 5.7	9.6 ± 4.6	3.9 ± 2.9	54.6 ± 23.5
*P* value	**0.004**	0.117	**0.016**	0.104	0.289	**0.019**

*Note*: The numbers in bold type indicate statistical significance.

### Personality strengths, personality disorder and the general neurotic syndrome

As might have been predicted, those with a baseline diagnosis of personality disorder using the ICD‐11 system had generally lower personality strengths, but the differences were not great, and the strengths of emotional toughness and discernment were generally similar except in those with moderate or severe personality disorder (Table 4). Those with a diagnosis of the general neurotic syndrome (GNS ≥ 6) had lower strengths in all elements, particularly in emotional toughness and independence compared to those without the syndrome.

**TABLE 4:** Relationship between personality strengths and baseline personality disorder and general neurotic syndrome status

Personality strengths: Mean ± SD						
Baseline status	Forcefulness	Emotional toughness	Cautiousness	Independence	Discernment	Overall
GNS score						
<6: 60	17.9 ± 7.2	22.7 ± 11.3	14.6 ± 7.2	12.1 ± 5.2	4.4 ± 3.1	69.9 ± 27.2
≥6: 28	15.1 ± 7.0	17.1 ± 10.0	12.1 ± 6.8	10.0 ± 4.7	4.0 ± 3.1	56.7 ± 26.2
*P* value	0.089	**0.028**	0.135	0.069	0.580	**0.035**
PD status						
None: 35	20.2 ± 6.2	24.0 ± 10.3	16.2 ± 6.9	12.7 ± 4.9	4.9 ± 3.0	75.4 ± 25.2
Difficulty: 16	16.8 ± 7.6	17.3 ± 11.5	11.2 ± 8.2	10.6 ± 5.7	4.1 ± 2.5	59.3 ± 31.2
Simple: 24	15.4 ± 6.9	21.3 ± 10.4	14.5 ± 6.1	11.5 ± 4.6	4.0 ± 3.3	64.9 ± 23.9
Moderate/severe: 12	11.5 ± 6.6	15.4 ± 12.4	8.6 ± 5.1	8.3 ± 4.8	3.0 ± 3.8	45.9 ± 25.5
*P* value	**0.001**	0.061	**0.004**	0.059	0.282	**0.008**

*Note*: The numbers in bold type indicate statistical significance.

### Personality strengths, suicidal behaviour and service contacts

The strongest associations were found between personality strengths and suicidal behaviour. Greater personality strengths in each of the five categories were significantly associated with fewer suicidal attempts at 30 years. At both 12 and 30  years those with no suicidal attempt was associated with high personality strength score of most categories (Table 5). Those who had little or no suicidal behaviour over time had the greatest personality strengths. Lower personality strengths were generally associated with unstable suicide behaviour. The association between personality strengths and social worker contacts was only demonstrated at 12 years, but lower personality strengths indicated increased use of the social services over time. Those with lower strengths, in particular with less forceful consideration and independence, were more likely to attend day care, and those with low forceful considerateness and cautiousness were also more likely to be in custody (Table 5).

**TABLE 5:** Suicidal behaviour and service contacts by personality strengths

	Personality strengths: Mean ± SD					
Characteristics	Forcefulness	Emotional toughness	Cautiousness	Independence	Discernment	Overall
Suicide attempts: *N* (30 years)						
None: 71	18.3 ± 6.8	23.2 ± 10.7	15.1 ± 7.1	12.3 ± 4.9	4.8 ± 3.2	71.5 ± 26.3
1–2 times: 12	13.9 ± 7.4	12.5 ± 7.5	11.1 ± 5.0	8.3 ± 4.3	3.0 ± 2.1	48.5 ± 18.3
3 times and more: 6	8.7 ± 3.8	9.5 ± 6.9	5.2 ± 3.4	6.7 ± 2.9	1.2 ± 1.5	32.0 ± 11.8
*P* value	**0.001**	**0.000**	**0.001**	**0.001**	**0.006**	**0.000**
*N*: (12 years)						
None: 70	18.5 ± 6.7	22.9 ± 10.8	15.4 ± 7.0	11.9 ± 5.2	4.6 ± 3.1	70.9 ± 27.2
1–2 times: 13	11.1 ± 5.9	12.6 ± 8.5	8.6 ± 4.9	8.6 ± 3.8	2.7 ± 2.2	44.3 ± 16.6
3 times and more: 6	12.8 ± 7.8	14.8 ± 10.6	7.3 ± 3.5	11.3 ± 4.8	4.2 ± 3.4	51.3 ± 21.5
*P* value	**0.001**	**0.003**	**0.000**	0.093	0.130	**0.002**
Change: *N*						
No change: 67	18.4 ± 6.9	23.2 ± 10.9	15.5 ± 6.9	12.2 ± 5.1	4.6 ± 3.2	71.5 ± 27.2
Increased attempts: 10	12.9 ± 7.2	11.9 ± 7.7	9.5 ± 6.3	7.0 ± 4.1	2.2 ± 2.3	43.0 ± 21.5
Reduced attempts: 12	12.9 ± 6.4	15.3 ± 9.2	8.4 ± 4.5	10.9 ± 4.1	4.0 ± 2.8	52.3 ± 16.7
*P* value	**0.006**	**0.001**	**0.000**	**0.009**	0.063	**0.001**
Social worker contacts: *N* (30 years)						
None: 79	17.7 ± 7.0	21.6 ± 11.1	14.3 ± 7.3	11.7 ± 4.9	4.4 ± 3.2	67.7 ± 27.1
1–5 times: 3	11.0 ± 3.5	13.7 ± 7.5	8.7 ± 5.9	5.7 ± 1.2	3.3 ± 1.5	40.0 ± 8.5
6 times and above: 7	12.9 ± 8.6	15.3 ± 11.3	11.7 ± 5.6	10.1 ± 6.7	3.6 ± 3.0	53.7 ± 28.1
*P* value	0.076	0.184	0.289	0.097	0.701	0.107
*N* (12 years)						
None: 75	17.9 ± 7.0	22.3 ± 10.6	14.9 ± 6.9	11.9 ± 5.0	4.4 ± 3.1	69.1 ± 26.8
1–5 times: 9	11.7 ± 5.8	14.3 ± 9.3	10.3 ± 7.1	7.6 ± 3.6	3.2 ± 3.6	46.7 ± 22.3
6 times and above: 5	13.8 ± 7.7	10.6 ± 13.6	6.0 ± 3.7	10.4 ± 6.3	5.0 ± 3.1	48.4 ± 27.3
*P* value	**0.025**	**0.012**	**0.007**	**0.042**	0.512	**0.021**
Change: *N*						
No change: 69	18.4 ± 6.8	22.7 ± 10.6	15.0 ± 7.0	12.2 ± 4.7	4.4 ± 3.1	70.6 ± 26.1
Increased contact: 8	11.9 ± 7.7	16.3 ± 10.3	12.0 ± 5.1	8.5 ± 6.4	3.4 ± 2.8	50.0 ± 27.0
Reduced contact: 12	12.6 ± 6.1	13.3 ± 11.1	8.6 ± 6.9	8.6 ± 4.7	4.1 ± 3.5	47.6 ± 24.6
*P* value	**0.003**	**0.010**	**0.010**	**0.014**	0.653	**0.005**
Day centre care: *N* (30 years)						
None: 85	17.5 ± 7.0	21.5 ± 10.9	14.2 ± 7.1	11.7 ± 5.0	4.4 ± 3.1	67.3 ± 26.8
Yes: 4	7.3 ± 3.5	7.3 ± 5.6	7.0 ± 3.7	5.5 ± 1.7	1.5 ± 1.7	31.3 ± 7.0
*P* value	**0.004**	**0.011**	**0.047**	**0.016**	0.067	**0.009**
*N* (12 years)						
None: 63	17.9 ± 6.9	21.8 ± 10.9	14.3 ± 7.2	12.0 ± 4.9	4.5 ± 3.1	68.6 ± 26.6
Yes: 20	13.1 ± 6.2	17.0 ± 10.7	12.0 ± 6.3	8.6 ± 4.5	3.3 ± 2.8	52.4 ± 23.6
*P* value	**0.007**	0.091	0.208	**0.007**	0.113	**0.017**
Change: *N*						
No change: 65	17.7 ± 6.9	21.5 ± 10.8	13.9 ± 7.3	11.9 ± 4.9	4.4 ± 3.0	67.7 ± 26.5
Increased use: 1	3.0 ± n/a	1.0 ± n/a	11.0 ± n/a	4.0±/n/a	0 ± n/a	22.0 ± n/a
Reduced use: 17	13.9 ± 6.4	18.4 ± 10.9	13.1 ± 6.0	9.1 ± 4.7	3.5 ± 2.9	55.6 ± 24.3
*P* value	**0.018**	0.113	0.850	**0.039**	0.194	0.066
In custody: *N* (30 years)						
None: 84	17.5 ± 7.1	21.3 ± 11.1	14.3 ± 7.2	11.6 ± 5.1	4.4 ± 3.1	67.3 ± 27.2
Yes: 5	10.0 ± 3.9	12.8 ± 7.8	7.4 ± 1.8	7.8 ± 3.5	2.0 ± 1.9	39.0 ± 10.0
*P* value	**0.022**	0.095	**0.035**	0.100	0.091	**0.024**
*N* (12 years)						
None: 84	17.4 ± 7.1	21.5 ± 11.0	14.4 ± 7.1	11.5 ± 5.1	4.3 ± 3.1	67.0 ± 27.3
Yes: 5	11.0 ± 6.8	10.8 ± 8.6	6.2 ± 3.3	9.8 ± 4.4	4.0 ± 2.6	44.6 ± 19.1
*P* value	0.051	**0.037**	**0.012**	0.466	0.837	0.075
Change: *N*						
No change: 81	17.8 ± 6.9	21.9 ± 10.9	14.6 ± 7.0	11.7 ± 5.1	4.5 ± 3.1	68.4 ± 26.8
Increased custody: 4	9.3 ± 4.1	11.3 ± 8.1	7.5 ± 2.1	7.3 ± 3.8	1.3 ± 0.9	35.3 ± 6.4
Reduced custody: 4	10.5 ± 7.7	8.8 ± 8.4	6.0 ± 3.7	9.8 ± 5.1	3.8 ± 3.0	42.3 ± 21.2
*P* value	**0.010**	**0.013**	**0.013**	0.183	0.123	**0.011**

*Note*: The numbers in bold type indicate statistical significance.

### Personality strengths and clinical symptomatology

The influence of personality strengths and clinical symptoms is shown in Table 6. All clinical measures demonstrated that higher clinical symptom scores were associated with lower personality strength scores, which were generally greater at 30 years than at 12 years of follow‐up. The one exception was the self‐rated HADS‐A (anxiety score) which showed no important differences over the 18 years between 12 and 30 years (Table 6).

**TABLE 6:** Pearson's correlation between personality strengths and clinical symptomatology

Personality strengths						
	Forcefulness	Emotional toughness	Cautiousness	Independence	Discernment	Overall
CPRS						
At 12 years (*N* = 89)	−0.457***	−0.422***	−0.523***	−0.263*	−0.169	−0.448***
At 30 years (*N* = 88)	−0.557***	−0.670***	−0.607***	−0.607***	−0.390***	−0.690***
Change over 18 years	−0.198	−0.368***	−0.198	−0.425***	−0.273*	−0.353**
MADRAS						
At 12 years (*N* = 89)	−0.467***	−0.391***	−0.502***	−0.278**	−0.219*	−0.446***
At 30 years (*N* = 88)	−0.550***	−0.690***	−0.613***	−0.616***	−0.403***	−0.702***
Change over 18 years	−0.052	−0.267*	−0.082	−0.290**	−0.154	−0.213*
BAS						
At 12 years (*N* = 89)	−0.299**	−0.335**	−0.395***	−0.164	−0.075	−0.317**
At 30 years (*N* = 88)	−0.525***	−0.684***	−0.600***	−0.511***	−0.422***	−0.666***
Change over 18 years	−0.258*	−0.397***	−0.259*	−0.345**	−0.337**	−0.379***
HADS‐D						
At 12 years (*N* = 89)	−0.503***	−0.467***	−0.563***	−0.387***	−0.270*	−0.532***
At 30 years (*N* = 88)	−0.477***	−0.650***	−0.609***	−0.564***	−0.361**	−0.684***
Change over 18 years	−0.204	−0.211*	−0.117	−0.156	−0.191	−0.211*
HADS‐A						
At 12 years (*N* = 89)	−0.284**	−0.346**	−0.425***	−0.257*	−0.136	−0.358**
At 30 years (*N* = 88)	−0.594***	−0.535***	−0.531***	−0.417***	−0.322**	−0.559***
Change over 18 years	−0.054	−0.147	−0.008	−0.144	−0.071	−0.111

* *p* < 0.05.

** *p* < 0.01.

*** *p* < 0.001.

The change in clinical symptomatology was defined in the same way as that of the SFQ and NDOS scales. All clinical measures apart from HADS‐A showed an association between improved clinical symptoms (reduced score) and greater personality strengths, with overall PASP score, and with emotional toughness as well as independence in particular. The BAS (Brief Anxiety Scale) (Tyrer et al., 1984) measure was the most responsive to the impact of personality strengths in improving clinical symptoms, followed by the CPRS (Comprehensive Psychopathological Rating Scale) measure.

## DISCUSSION

This study has found associations between higher personality strength scores and better social function, better neurotic disorder condition, better clinical outcomes and less use of services overall. Of especial interest is the link between suicidal behaviour and low personality strengths, which are of particular interest. There are some other suggestions that such strengths can help to prevent suicidal thinking (Kim et al., 2018; Nagy et al., 2021; Williamson et al., 2019) and they are worthy of closer study.

Over time, personality strengths were also associated with improved social function and clinical outcomes as well as stabilised service uses and behaviour. These data illustrate that the inter‐relationships between positive and negative personality are complex and the notion of universal blanket pathology in personality disorder cannot be supported (Widiger, 2016; Wilson et al., 2017).

But the study has important limitations. First, the personality strengths were not measured at the beginning of the project; hence, their impacts on any other measures were either cross sectional (at 30 years) or retrospective (at baseline or 12 years). No causal effects could therefore be justified from the study. Second, as it is known that personality disorder status changes over time (Yang et al., 2021; Zanarini et al., 2003), there are no data on changes in personality strengths over a long time period. The conclusions that can be drawn can therefore only be provisional. But as personality disorder changes over time, it is reasonable to infer that personality strengths may do so also.

### Is the separation of personality strengths into groups valid?

The personality strengths by elements were highly correlated with Pearson's correlation coefficients in the range of 0.472–0.721, which is similar to most psychometric scales, hinting at an underlying co‐varying structure. In most situation, separate analysis of each element was sufficient, which has been supported in the results. However, the groups in the study maybe different from other based on different sample. Further psychometric analysis based on multiple samples is required to confirm and validate the groups identified from the CPAS. An abbreviated version of the CPAS derived from the main interview schedule and administered by an interview or self‐rating is shown (supporting information).

### Are personality strengths the opposite of personality disorder?

What is clear is that the obverse of personality disorder is not a simple reversal of the negative aspects of personality disturbance. It is a complex mix of abilities and strengths that interleave with personality disturbance. In some instances, strengths can be developed from disordered personalities; the experience of disorder can give insights into ways of off‐setting or changing it, and the results in the emotionally tough domain of the strengths scale in those with personality disorder (Table 4) appear to illustrate this.

The presence of personality strengths may also have an impact on treatments for many mental disorders and explain differences in the results of studies. For example, a recent study showing that personality disorder had a negative impact on the outcome of treatment of depression and anxiety in the Improved Access to Psychological Treatments (IAPT) programme in the United Kingdom (Goddard et al., 2015) was not replicated in a subsequent Australian study using internet CBT (Mahoney et al., 2021). The populations accessed by these different methods may be a consequence of different personality strengths.

A personality problem can be a personality strength at the same time. In another study of the treatment of health anxiety in medical patients with an adapted form of CBT, those with personality disorder had significantly better outcomes compared with non‐personality disordered patients, and the findings suggested that better adherence to the treatment programme and lower drop‐outs in the personality disordered group (who had prominent anankastic and anxious domain traits) was the main reason for this (Tyrer, Wang, et al., 2021).

Although the several limitations of the study prevent firm conclusions from being drawn, the findings hint at an important part of personality structure that has hitherto been only partly appreciated. It is a subject that would repay further enquiry, especially in studies of suicidal behaviour.

## CONFLICT OF INTEREST

None of the authors has any conflict of interest to declare.

## ETHICS STATEMENT

Ethical approval for follow‐up was granted by Northampton Research Ethics Committee (12/EM/0331).

## Supporting information


**Data S1.** Abbreviated version of Comprehensive Personality Assessment Scale in self‐report or interview format

## Data Availability

The study data are available from Professor Min Yang.
