# Supplementary material for: The recording of personality strengths: An analysis of the impact of positive personality features on the long‐term outcome of common mental disorders
Source: Personal Ment Health. 2022 May 9;16(2):120–9. doi: 10.1002/pmh.1548 (PMC9287073; doi:10.1002/pmh.1548)
Supplement: Supplementary file 1 — Data S1. Abbreviated version of Comprehensive Personality Assessment Scale in self‐report or interview format [file PMH-16-120-s001.pdf]

Abbreviated Personality Strengths Scale (shortened version of PASP)

I would now like to ask you some questions about your personality strengths. There are five questions and in answering them please take into account all the times when these strengths may have been present. (The interviewer has to assess both presence and outcome of strength over time and may prefer to administer the scale rather than rely on self-completion)

A. Are you a person who has the strength to follow things through and can do this whilst still taking into account the feelings of others? Have there been occasions in your life when you have insisted on following your own path and persuaded others that this was the right thing to do? (Ask for examples from personal life, occupation and in relationships)

no evidence of strength	0
very slight evidence of strength but no evidence in practice	1
minor evidence of strength but with small effects only	2
some evidence of strength with greater evidence of positive outcome	3
clear evidence of strength in more than one area	4
definite evidence of strength in more than one area with good outcome	5
definite evidence of strength in several areas with very clear positive outcomes	6
pronounced evidence of strength with both short and long-term positive results	7
persistent demonstration of strength throughout life fully accepted by others	8

(This is the Abraham Lincoln strength)

B. Are you a person who can use your knowledge of your own feelings to overcome emotional problems? People like this can not only help themselves but also others as they well understand the feelings and distress of others. (Ask for examples from personal life, occupation and particularly in relationships)

no evidence of strength	0
very slight evidence of strength but not successfully put into practice	1
minor evidence of strength but with occasional examples of success	2
some evidence of strength but put into practice only occasionally	3
clear evidence of strength with success in several relationships	4

definite evidence of strength on many occasions	5
pronounced evidence of strength with evidence of self-help and well as others	6
pronounced evidence of strength accompanied by long-term positive results	7
persistent demonstration of strength with good outcomes over time	8

(The Princess Diana strength)

C. Are you a person who has found it helpful to be careful and cautious before making decisions in life? Has this been appreciated by others as well. (Add examples of prudence and care – eg not rushing to spend money that arrives unexpectedly, planning in the longer term, playing a waiting game)

no evidence of strength	0
very slight evidence of strength but not enough to have any value to date	1
minor evidence of strength but with little in the way of results	2
some evidence of strength but limited results	3
clear evidence of strength with good examples of caution being successful	4
definite evidence of strength over time with long-term benefits	5
definite evidence of strength with pronounced benefit	6
persistent evidence of strength with very clear benefit to many others	7
persistent demonstration of strength over a long period with great success	8

(This is the Fabius Cunctator strength)

D. Are you a person who can thing on your own and make good decisions without the help of others? Would you say you were an independent thinker? (Add examples: how have independent attitudes helped in life?)

no evidence of strength	0
very slight evidence of strength but only slight evidence of any results	1
minor evidence of strength but independence has been of limited value	2
some evidence of strength with some notable but small gains	3
clear evidence of strength with success related to circumscribed areas	4
definite evidence of strength with successful outcomes that have helped	5

definite evidence of strength which has clearly reinforced well-being	6
persistent demonstration of strength which has helped greatly over life course	7
pronounced evidence of strength accompanied by impressive long-term results	8

(This is the Steve Job strength)

E. Are you able to work out what others are thinking and doing so it helps you in life? Can you see the bigger picture and plan ahead? (Add examples of how this greater understanding has helped the person and others)

no evidence of strength	0
very slight evidence of strength but only dimly exercised	1
some evidence of strength with occasional positive results of minor degree	2
clear evidence of strength with success in helping self and occasional others somewhat	3
definite evidence of strength with long-term success in helping self and others	4
definite evidence of strength over time with bigger impact on others as well as self	5
pronounced evidence of strength accompanied by long-term positive value to many	6
persistent demonstration of strength with great evidence of success over time	7
life-long evidence of strength that has been of great benefit to self and others	8

(This is the Angela Merkel strength)

Abraham Lincoln strength = forceful considerateness

Princess Diana strength = emotional toughness

Fabius Cunctator strength = cautiousness

Steve Job strength = independence

Angela Merkel strength = discernment

At this stage it is not possible to assign appropriate thresholds to each strength
